# Adult granulosa cell tumor of the testis with malignant tendency: A case report with genetic analysis using high-throughput sequencing

**DOI:** 10.1097/MD.0000000000034523

**Published:** 2023-08-11

**Authors:** Lili Deng, Jingjing Zeng, Jin Feng Qiu, Li Hua Yang, Jie Ma

**Affiliations:** a Department of Oncology, The First Affiliated Hospital of Guangxi Medical University, Nanning, Guangxi Province, China; b Department of Pathology, The First Affiliated Hospital of Guangxi Medical University, Nanning, Guangxi Province, China.

**Keywords:** adult granulosa cell tumor, CDKN2A, genetic, H3F3A, TERT, testis

## Abstract

The adult granulosa cell tumor of the testis is a rare sex-cord/stromal tumor, with a potentiality for late recurrence and metastasis. Because of its rarity, this tumor is poorly understood, particularly in terms of its molecular features. As a result, it is necessary to register each occurrence in order to study the evolution of this rare malignancy and develop therapeutic strategies.

**Methods:**

A 50-year-old man discovered a painless right testicular mass unexpectedly, and the mass steadily expanded for 2 months. Ultrasonography showed a 5.2 cm × 4.0 cm × 3.6 cm mass in the right testicle. A right radical orchiectomy was performed on September 7, 2016. The pathologic diagnosis was a testicular adult granulosa cell tumor. The post-computed tomography scans and bone scintigraphy ruled out distant metastases. A high-throughput sequencing of 520 cancer-related genes revealed FOXL2 C134W, CDKN2A E87Gfs*24, TP53 S183*, TERT c.-124C > T, and H3F3A K28R mutations in this case. Because the patient stated he would be unable to return to the hospital for a follow-up appointment on time, he elected to have 4 cycles of adjuvant chemotherapy BEP (bleomycin, etoposide, and cisplatin) after the right radical orchiectomy.

**Results:**

The patient has not had a clinical recurrence or metastasis in 6 years.

**Conclusion:**

Surgery together with adjuvant chemotherapy may be useful treatment options for these individuals with malignant tendencies who are unable to visit the hospital for a follow-up appointment on time. Adult testicular granulosa cell tumors have a relatively complex genetic profile; their etiology is linked to a number of common driver genes, including TERT, CDKN2A, TP53, and H3F3A.

## 1. Introduction

The adult granulosa cell tumor (AGCT) of the testis is extremely rare. <80 cases have been described since the first occurrence was reported in 1952.^[1]^The pathophysiology, prognostic variables, and optimum management issues of testicular AGCTs are yet unknown. With the advent of high-throughput sequencing technology, it is now possible to expose the genomic properties of this tumor, allowing us to better comprehend its biology.

## 2. Case presentation

**Figure 1.**
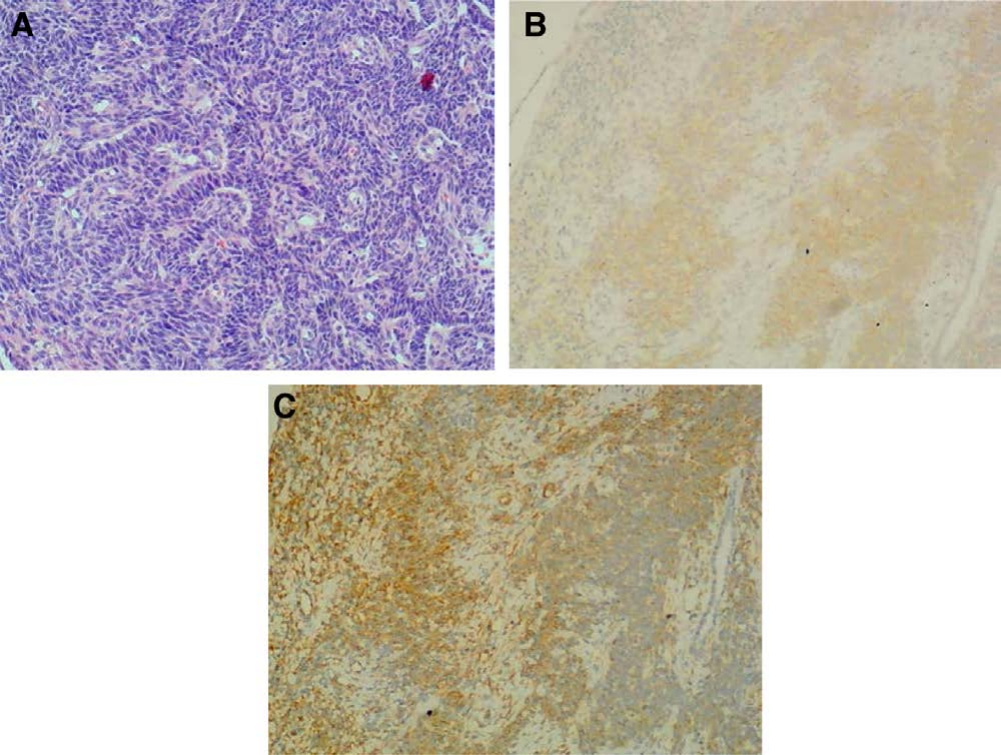
(A) Hematoxylin and eosin staining at 10 × magnification. (B) Inhibin staining at 10 × magnification. (C) Vimentin staining at 10 × magnification.

A 50-year-old man was hospitalized for a 2-month history of a right testicular lump in July 2016. Upon further investigation, he revealed a history of chronic gastritis as well as being a hepatitis B virus carrier. Physical examination revealed a painless, palpable, well-defined mass. An ultrasound revealed a 5.2 cm × 4.0 cm × 3.6 cm lump in the right testicle. A laboratory examination indicated no abnormalities. On September 7, 2016, a right radical orchiectomy was performed. Histopathological examination revealed granulosa cell tumor with characteristic morphologic features (Fig. 1A), but no invasion to surgical margins, spermatic cord, or epididymis. Immunohistochemical staining of the tumor cells demonstrated positivity for inhibin (Fig. 1B), vimentin (Fig. 1C), and negativity for CD117, CD30, CK, CK8, epithelial membrane antigen, placental alkaline phosphatase, and S-100 protein. These findings strongly suggested an AGCT diagnosis. A high-throughput sequencing of 520 cancer-related genes was found to examine the histopathologic character and genetic changes of this patient, the results are described in Table 1. This patient’s post-computed tomography scan and bone scintigraphy ruled out distant metastases. Although neither postoperative adjuvant chemotherapy or radiation therapy were indicated, the patient was required to return to the hospital on time for follow-up consultations. However, the patient continued to be anxious since he indicated that he could not receive follow-up on time. After consulting with a multidisciplinary team (including a radiation oncologist, a medical oncologist, a surgeon, a radiologist, and a pathologist), the patient decided to undergo 4 cycles of adjuvant BEP (bleomycin, etoposide, and cisplatin) from October 2016 to July 2017.The patient tolerated the medication well, the main negative effect was myelosuppression. He has now been followed for 6 years, and no evidence of clinical recurrence or metastasis have been identified yet.

**Table 1:** Genome analysis of a testicular AGCT.

Gene name	Variant information	Position	Variation in abundance
FOXL2	C134W	exon1	8%
TERT	c. −124C > T	-	25%
CDKN2A	E87Gfs*24	exon2	2%
TP53	S183*	exon5	5%
H3F3A	K28R	exon2	35%

AGCT = adult granulosa cell tumor.

*indicates termination codon for mutation.

## 3. Discussion and conclusion

According to the literature, testicular AGCTs grow slowly but have a malignant propensity to form distant metastases in 20% of instances, and those metastases frequently emerge several years after the initial diagnosis.^[2]^Patients outcomes are exceedingly dismal after metastasis begins. The main challenge in treating patients with testicular AGCTs is evaluating the possibility for malignancy and the appropriate management concerns for the most aggressive cases. A few clinicopathological features suggestive of cancer have been proposed, such as tumor size > 4 or 5cm, mitoses > 30 per 10 HPF, age at diagnosis > 50 years, angiolymphatic invasion, necrosis, cellular atypia, and gynecomastia.^[3–5]^However, the only constant marker in predicting malignancy is a tumor size more than 4 or 5cm.^[6,7]^

In terms of treatment, there is no consensus for testicular AGCT. The primary treatment for AGCT is surgery.^[5]^ It is still unknown what function postoperative adjuvant chemotherapy and/or radiotherapy will play.^[3]^However, due of the late metastatic symptoms, a follow-up program for these individuals is necessary. Given our patient’s 2 clinicopathological risk indicators for malignancy (tumor size > 4cm, age at diagnosis > 50 years), he was deemed to have a relatively high risk of malignancy. Because the patient stated that he would be unable to come to the hospital on time for his follow-up, he elected to have adjuvant chemotherapy after the right radical orchiectomy. This patient is still free of local recurrence or metastases after 6 years. Surgery combined with adjuvant chemotherapy could be helpful treatment measures for these patients who have a malignant predisposition but are unable to follow-up on time.

To present, only few genome sequencing studies on testicular AGCT cases have been completed, and the molecular characteristics characterizing the disease’s biological behaviors are still unknown. Previous research has indicated that testicular AGCT is molecularly diverse, with ATM, TP53, and NRAS mutations being implicated in particular cases.^[6]^We discovered that many common driver genes, including TERT c.-124C > T, CDKN2A E87Gfs*24, and H3F3A K28R mutations, were also linked to the etiology of testicular AGCTs in our investigation. To the best of our knowledge, these gene mutations have never been described in testicular AGCT.

The FOXL2 C134W mutation is the primary cause of ovarian AGCT oncogenesis. Because more than 95% of patients carry this mutation, the FOXL2 C134W mutation has been identified as a pathognomonic characteristic for ovarian AGCT.^[8]^The FOXL2 C134W mutation was also found in testicular AGCTs, but with low expression rates (20%, 4 of 20).^[2,6,9,10]^ Because this mutation test was only performed on a small number of testicular AGCT patients, the significance of the FOXL2 C134W mutation in this condition is currently unknown. Except for 1 case, the diameters of the other 2 testicular AGCTs with FOXL2 C134W mutations in the literature were 3cm and 2.5cm, respectively.^[6,10]^They exhibited no signs of malignancy. As a result, it appears that the FOXL2 C134W mutation has no effect on the progression of testicular AGCT from benign to malignant.

TERT c. −124C > T (also known as C228T) is a harmful somatic mutation that promotes cell immortalization and cancer by increasing TERT expression and telomerase activity. TERT c. −124C > T has been documented in over 50 human malignancies, with many TERT-mutated patients having significantly lower overall survival than wild-type patients.^[11–13]^Recent research in ovarian AGCTs have found that the frequency of TERT c. −124C > T mutations is much higher in recurrent ones (41–67%) than in primary ones (20–29%).^[14,15]^Furthermore, 1 study discovered TERT c. −124C > T alterations after an ovarian AGCT developed into a high-grade sarcoma.^[16]^CDKN2A E87Gfs*24 and TP53 S183* were 2 more significant genetic alterations discovered in our case. CDKN2A and TP53 are cell cycle genes that play critical roles in cell growth and apoptosis.^[17,18]^In the presence of oncogenic driver mutations, mutated CDKN2A and TP53 are unable to promote cell cycle arrest or apoptosis. According to several research, CDKN2A and/or TP53 mutations are believed to be early molecular events in the malignant evolution of malignancies, and are associated with more aggressive disease and poor outcome.^[19,20]^In a study of a subset of ovarian AGCT cases, recurrences included additional genetic abnormalities such as TP53 mutations and CDKN2A/B homozygous deletions that were not found in primary cases.^[15]^Another study discovered 1 testicular AGCT patient with multiple malignancy-like characteristics, including large size (9.5cm), cellular atypia, high mitotic activity (107 mitoses/10 HPF), and invasive development. In addition, the patient had molecular evidence of TP53 inactivation.^[6]^An H3F3A K28R mutation was also found in this case. H3F3A encodes H3.3 core histone proteins, and H3F3A mutations have largely been identified in brain and bone malignancies.^[21]^It is thought to be one of the most important predictors of poor prognosis in juvenile gliomas.^[22]^However, no cases of this mutation have been recorded in urogenital system malignancies. Based on the findings, we hypothesize that TERT, CDKN2A, TP53, and H3F3A mutations may play a role in the transition of benign to severe testicular AGCT.

In conclusion, we examined a rare instance of testicular AGCT with 2 clinicopathological characteristics suggestive of malignancy using genomic analysis. We discovered that the genomic profile of testicular AGCT is relatively complex, with a varied number of common driver gene mutations linked with this pathogenesis, including TERT, CDKN2A, and TP53 alterations. Larger investigations are required to further investigate and determine the clinical significance of those genetic alterations in testicular AGCT.

## Author contributions

**Conceptualization:** Lili Deng.

**Supervision:** Jie Ma.

**Writing – original draft:** Lili Deng.

**Writing – review & editing:** Jie Ma, Jingjing Zeng, JinFeng Qiu, Li Hua Yang.
